# Functional Attributes and Anticancer Potentialities of Chico (*Pachycereus Weberi*) and Jiotilla (*Escontria Chiotilla*) Fruits Extract

**DOI:** 10.3390/plants9111623

**Published:** 2020-11-22

**Authors:** Luisaldo Sandate-Flores, Eduardo Romero-Esquivel, José Rodríguez-Rodríguez, Magdalena Rostro-Alanis, Elda M. Melchor-Martínez, Carlos Castillo-Zacarías, Patricia Reyna Ontiveros, Marcos Fredy Morales Celaya, Wei-Ning Chen, Hafiz M. N. Iqbal, Roberto Parra-Saldívar

**Affiliations:** 1 Tecnologico de Monterrey, School of Engineering and Sciences, Centro de Biotecnología FEMSA, Avenida Eugenio Garza Sada 2501, Monterrey 64849, Mexico; luisaldosf@gmail.com (L.S.-F.); romeroe.ing@gmail.com (E.R.-E.); jrr@tec.mx (J.R.-R.); magda.rostro@tec.mx (M.R.-A.); elda.melchor@tec.mx (E.M.M.-M.); carloscastilloz@tec.mx (C.C.-Z.); 2 Universidad Iberoamericana, Puebla, Avenida Tres Oriente, 615, 6, Centro, Puebla C.P. 72000, Mexico; direccion@rab-gts.com; 3 Universidad Tecnológica de los Valles Centrales de Oaxaca, Oaxaca 71270, Mexico; mfmorcel@gmail.com; 4 School of Chemical and Biomedical Engineering, College of Engineering, Nanyang Technological University, 62 Nanyang Drive, Singapore 637459, Singapore; wnchen@ntu.edu.sg

**Keywords:** pachycereus weberi, escontria chiotilla, antioxidant activity, phenolic compounds, betalains, food composition, food analysis, cytotoxicity

## Abstract

Mexico has a great diversity of cacti, however, many of their fruits have not been studied in greater depth. Several bioactive compounds available in cacti juices extract have demonstrated nutraceutical properties. Two cactus species are interesting for their biologically active pigments, which are chico (*Pachycereus weberi* (*J. M. Coult.) Backeb*)) and jiotilla (*Escontria chiotilla* (*Weber) Rose*)). Hence, the goal of this work was to evaluate the bioactive compounds, i.e., betalains, total phenolic, vitamin C, antioxidant, and mineral content in the extract of the above-mentioned *P. weberi* and *E. chiotilla*. Then, clarified extracts were evaluated for their antioxidant activity and cytotoxicity (cancer cell lines) potentialities. Based on the obtained results, Chico fruit extract was found to be a good source of vitamin C (27.19 ± 1.95 mg L-Ascorbic acid/100 g fresh sample). Moreover, chico extract resulted in a high concentration of micronutrients, i.e., potassium (517.75 ± 16.78 mg/100 g) and zinc (2.46 ± 0.65 mg/100 g). On the other hand, Jiotilla has a high content of biologically active pigment, i.e., betaxanthins (4.17 ± 0.35 mg/g dry sample). The antioxidant activities of clarified extracts of chico and jiotilla were 80.01 ± 5.10 and 280.88 ± 7.62 mg/100 g fresh sample (DPPH method), respectively. From the cytotoxicity perspective against cancer cell lines, i.e., CaCo-2, MCF-7, HepG2, and PC-3, the clarified extracts of chico showed cytotoxicity (%cell viability) in CaCo-2 (49.7 ± 0.01%) and MCF-7 (45.56 ± 0.05%). A normal fibroblast cell line (NIH/3T3) was used, as a control, for comparison purposes. While jiotilla extract had cytotoxicity against HepG2 (47.31 ± 0.03%) and PC-3 (53.65 ± 0.04%). These results demonstrated that Chico and jiotilla are excellent resources of biologically active constituents with nutraceuticals potentialities.

## 1. Introduction

**Figure 1:**
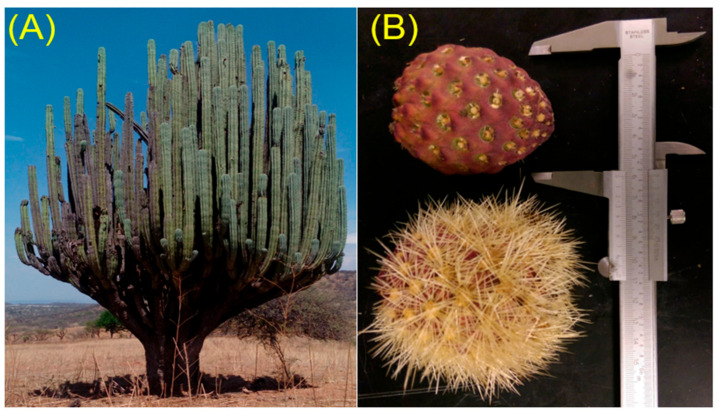
(**A**) *Pachycereus weberi* plant and (**B**) *P. weberi* fruit without prickles (top) with prickles (bottom).

Mexico has a great diversity of cacti [1], but many of the fruits from these plants have not been studied. Cacti fruits have health benefits such as anticlastogenic capacity [2], hepatoprotective effect [3], antimicrobial activity [4], notable antioxidant capacity [5,6], and anticancer activity [7,8]. Currently, the scientific community is interested in two cacti fruits for their pigments, chico (*Pachycereus weberi (J. M. Coult.) Backeb*) and jiotilla (*Escontria chiotilla (Weber) Rose)*. *Pachycereus weberi* is typical in the south-central Mexico region, specifically the arid and semi-arid regions in Puebla, Guerrero, Morelos, and Oaxaca [1]. The fruit of *Pachycereus weberi* is better known as “chico fruit” and “tuna de cardón” [9] (Figure 1). *P Weberi* fruits are ellipsoidal, 6 to 7 cm in diameter [10]. In Santiago Quiotepec, Oaxaca, fruits of this species harvested and commercialized in local markets [11]. The fruit production is 509.3 ton in Tehuacán Valley México from *Pachycereus weberi* [12].

**Figure 2:**
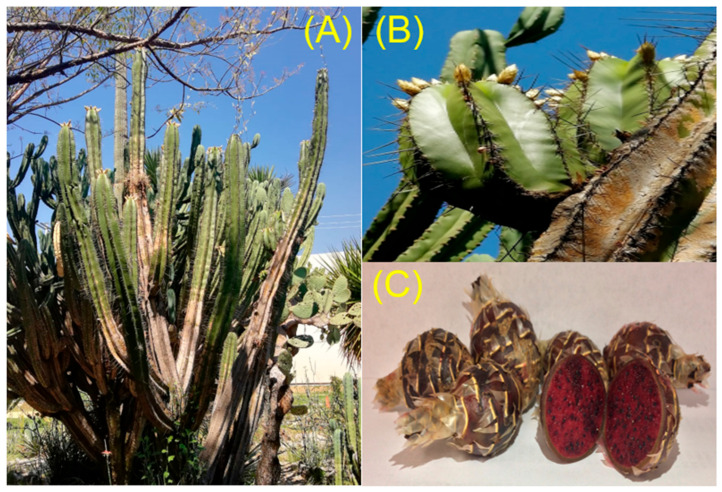
(**A**) *Escontria chiotilla* plant, (**B**) *E. chiotilla* flower, and (**C**) *E. chiotilla* fruit.

Nowadays, the knowledge about antioxidant activity and phenolic compounds of the fruit from the Mexican plant *Pachycereus weberi* is limited. *Escontria chiotilla,* a columnar cactus is found in Guerrero, Michoacán, Puebla, Oaxaca, and Morelos. The fruit of *Escontria chiotilla* has a red shell with the presence of scales, spherical, and are 5 cm in diameter (Figure 2). The period of growth of the fruit ranged 140 to 175 days [13] and is usually named as “chonostle” and “jiotilla” [14]. The production of this fruit is approximately 99.5 tons in the valley of Tehuacan [12] and is commercialized in local markets, and people from the community of Coxcatlan prepare ice cream and jellies for local trade [15]. Different studies have shown that the pigments present in the jiotilla are vulgaxanthin I, vulgaxanthin II, indicaxanthin, and betanin [16]. These pigments have been extracted using acidified mucilage and microencapsulated for subsequent characterization [17].

Based on the literature, cacti fruits have cytotoxic properties in cancer cell lines [7,8]. For instance, prickle pear juices (*Opuntia spp.*) have shown to produce decreasing viability of cancer cell in vitro, especially colon (HT29/Caco-2) and prostate cancer (PC-3) [7,8]. Indeed, these are promising results as around 70% of cancer deaths occur in low and middle incomes countries [18] and in 2018, 1.8 million colorectal and 1.28 million prostate cases were reported [18]. Furthermore, several studies have shown that fruits growth in semi-arid and arid lands present high mineral content [19,20]. This aspect is relevant as minerals are essential in metabolism and homeostatic of the human body, deficiency of minerals can lead to symptoms of common disorders and diseases as osteoporosis and anemia [21]. One of the essential micronutrients is zinc, and the deficiency of this restricts childhood growth and decreases resistance to infections [22]. In the world, around 20% of children under five years of age are stunted [23]. The principal food with a high concentration of zinc are oysters, red meat, and poultry [24]. The food mentioned before may be more challenging to access for low-income populations [25]. Oaxaca, Guerrero, and Puebla are Mexican states with low-income populations [26]. It is crucial to find other sources of zinc that are accessible to all communities.

Studies on potentialities of cacti fruits have gradually increased in recent years, but there is still a lack of information regarding antioxidant capacity, metal content, or phytochemical content of *P. weberi* and *E. chiotilla* fruits pulp and juice extracts. Therefore, this study aimed to evaluate the physiochemical characteristic, antioxidant activities, and anticancer potentialities of *P. weberi* and *E. chiotilla* fruits pulp and/or juice extracts.

## 2. Materials and Methods

### 2.1. Chemicals

2,2-Diphenyl-L-picryl-hydrazyl (DPPH) (C_18_H_12_N_5_O_6_, Item D9132, Lot 115K1319) was obtained from Sigma-Aldrich (Steinheim, Germany). Methyl alcohol (CH_3_OH, Item MS1922, Lot 17020429) was obtained from Tedia High Purity Solvents (Fairfield, OH, USA). Folin&Ciocalteu’s Phenol Reagent (C_7_H_6_O_5_, Item F9252, Lot SHBD7847V) was obtained from Sigma Aldrich (St. Louis, MO, USA). Sodium Carbonate (Na_2_CO_3_, Item S7795, Lot BCBP4310V) was obtained from Sigma Aldrich (Steinheim, Germany). Gallic acid monohydrate (C_7_H_6_O_5_, Item 398225, Lot MKBD1204) was obtained from Sigma-Aldrich (Shanghai, China). Sodium chloride (NaCl, DEQS290000100, Lot CS180307-03) was obtained from Desarrollo de Especialidades Químicas, S.A. de C.V. (Nuevo Leon, Mexico). Potassium persulfate (K_2_S_2_O_8_, Item 216224, Lot MKBR1923V) was obtained from Sigma-Aldrich (St. Louis, MO, USA). Disodium phosphate (Na_2_HPO_4_, 35902, Lot 536405) obtained from Productos Quimicos Monterrey S.A. de C.V. (Nuevo Leon, Mexico). Potassium chloride (KCl, S32204, Lot 536161) obtained from Productos Químicos Monterrey S.A. de C.V. (Nuevo Leon, Mexico). 2,2′-Azino-bis(3-ethylbenzothiazoline-6-sulfonic acid) ABTS (C_18_H_24_N_6_O_6_S_4_, Item A9941, Lot SLBT7934) obtained from Sigma-Aldrich (St. Louis, MO, USA). Potassium phosphate monobasic (KH_2_PO_4_, Item P0662, Lot SLBL9298V) was obtained from Sigma-Aldrich (Tokyo, Japan). Nitric acid (HNO_3_, Item UN2031, Lot SCA7227127) was obtained from SPC Science (Baie-d’Urfé, QC, Canada). Ammonium acetate (CH_3_COONH_4_, Item 0596, Lot L15C82) was obtained from J. T. Baker (Estado de Mexico, Mexico). Milli-Q water purification system is used to obtain the water that was used to perform the procedures (Q-POD, Darmstadt, Germany). Glacial acetic acid (Item AE4001, Lot 1004007) was bought from Tedia (Fairfield, OH, USA). 2,4,6-Tris(2-pyridyl)-s-triazine (TPTZ) (C_18_H_12_N_6_, Item T1253, Lot BCBT8262) was bought from Sigma Aldrich (Buchs, Switzerland). Iron (lll) chloride hexahydrate (FeCl_3_·6H_2_O, ItemF2877, Lot MKBR8795V) was bought from Sigma Aldrich (Shanghai, China). Hydrochloric acid (HCl, Item 00636, Lot 71973) was bought from CTR Scientific (Nuevo Leon, Mexico). Gallic acid monohydrate (C_7_H_6_O_5_·H_2_O, item 398225, Lot MKBD1204) was bought from Sigma Aldrich (Shanghai, China). Caffeic acid (C_9_H_8_O_4_, item C0625, Lot 089K1114) was obtained from Sigma-Aldrich (Buch, Switzerland). p-Coumaric acid (C_9_H_8_O_3_, item C9008, Lot BCBN0412V) was bought from Sigma-Aldrich (United Kingdom). Violuric acid (C_4_H_3_O_4_·H_2_O, item 95120, Lot BCBN0412V) and fumaric acid (C_4_H_4_O_4_, item 47910, Lot BCBM2191V) were obtained from Sigma-Aldrich (Vienna, Austria).

### 2.2. Collection and Preprocessing of Chico and Jiotilla

A single batch of chico fruits (10 kg) was collected from a crop field in Ahuatlán (Puebla, México). The sampling area was situated between 18°34′ latitude (North) and 98°15′ longitude (West). The collected fruit samples were stored at 5 °C for 48 h and processed within 72 h of harvest from the plant. Whereas, a batch of jiotilla fruits (22 kg) was collected from a crop field in Santa María Zoquitlan, Tlacolula, Oaxaca, Mexico 16°33′ latitude (North) and 96°2315′ longitude (West). The collected fruit samples were stored at 5 °C for 12 h and processed within 24 h of harvest from the plant. As collected fruits of both plants, i.e., chico and jiotilla, were preprocessed by washing with tap water and Extran MA05 (Merck, Item 1400001403, Lot Mx1400005004, Estado de Mexico, Mexico). Then, the prickles were removed manually with care using a sterile blade. The electric extractor (Model TU05, Turmix ML, Estado de México) was used to prepare a seed-free pulp sample. This method was used since it is a quick way to eliminate the seeds. Subsequently, around 5 g of seed-free pulp samples were placed in 40 mL vials, 10 mL of deionized water was added, and then the vials were agitated for 15 min. The resultant mix was filtered (Whatman paper grade 4, 150 mm, Item 1009150, GE Healthcare Life Sciences, Little Chalfont, UK) under dark conditions to avoid compounds degradation. The extract obtained (40 mL) was placed in a 100 mL (chico fruit) or 50 mL (jiotilla) volumetric flask and used in the subsequent analysis. Different dilutions were made because a great content of antioxidant capacity was expected in chico fruit. Three extractions for each fruit were carried out following the same conditions.

### 2.3. Clarified Juice Extract Preparation

Seed-free pulp (30 g) from each fruit was placed in 50-mL polypropylene centrifuge tubes (Corning^®^, Tewksbury, MA, USA). The tube was subjected to centrifugation (4000× *g*, 4 °C, 10 min, Model SL 40R, Thermo Fisher Scientific, Langenselbold, Germany). The supernatant was filtered in the dark through 150 mm Whatman paper grade 4 (item 1009150, GE Healthcare Life Sciences, Little Chalfont, UK). The pellets were removed, and the clarified supernatant from the pulp of each fruit was used as a clarified juice extract.

### 2.4. Physiochemical Characterization

Weight of pulp and peel were determined with an analytical balance (OHAUS, model Scout Pro SP4001, China). Equatorial and polar diameters were measured with a caliper (Vernier from Truper, model H87). The pH was determined with Termo Fisher Scientific, ORION 3 STAR pH Benchtop (Singapore, Singapore). Total soluble solids (Brix) were determined with a refractometer HI96811 (HANNA, Smithfield, RI, USA), and moisture content (%) was obtained according to the gravimetric method [27]. A general proximate analysis was applied to the pulps, the method used was AOAC 1999 [28].

### 2.5. Betacyanins and Betaxanthins Quantification

A spectrophotometric method is used to determine the concentration of betacyanins and betaxanthins in the pulp of the fruit. The spectrophotometer used was Model DR 500 (Hach Lange GmbH, Düsseldorf, Germany), following the methods described by Sandate-Flores et al. [29]. For the dilution of the samples, water Milli-Q was preferred, the dilution was 1:10, and it was carried out in 5 mL volumetric flasks. The extinction coefficients used were the following: for betacyanin (E1% = 60,000 L mol^−1^ cm^−1^, λ = 540 nm) and for betaxanthin (E1% = 48,000 L mol^−1^ cm^−1^, λ = 480) [30]. The betalains concentration was showed in milligrams per gram (mg/g, dry weight). The procedure for determining moisture content is described in Section 2.4.

### 2.6. Vitamin C Quantification

The vitamin C quantification was performed based on the procedure described earlier by Santos et al. [31]. The sample was passed through a nylon filter of 0.20 mL (2 mL of the volumetric extract), placed in vials for HPLC. The HPLC-FLD system (Agilent 1200 HPLC, Santa Clara, CA, USA) was injected with 25 µL of the sample, for which a Zorbax Eclipse XDB C18 column was used (5 µm, 150 9 4.6 mm i.d.) (Agilent, Santa Clara, CA, USA). As eluent solvents, a solution of 10 mM ammonium acetate at a pH of 4.5 was used (phase A), and a methanol solution with 0.1% acetic acid was phase B. Using 80% of phase A, elution was carried out in isocratic mode for 7 min with a flow rate of 1 mL/min. For the measurement of ascorbic acid, the chromatograms were monitored at Exc = 280 and Em = 440 nm. A six-point calibration curve was made using an external standard of ascorbic acid (0.01 to 10 mg/L), the curve used for the quantification of vitamin C.

### 2.7. Antioxidant Activity

#### 2.7.1. Folin-Ciocalteu Method (Total Antioxidant Capacity)

The Folin-Ciocalteu colorimetric method was used to determine the total antioxidant capacity [32]. This colorimetric method was carried out following the procedure of Singleton et al. [33]. The analysis was performed directly in 96 well plates. Briefly, around 20 µL of the diluted sample was placed in Milli-Q water, then 100 µL of folin reagent at a concentration of 10% was added. After 5 min of the reaction period, around 80 µL of sodium carbonate at a concentration of 7.5% *w*/*v* was added. The above reaction mixture was then incubated at 37 °C for 90 min in the dark. Finally, the sample containing 96-well microplate was placed in a plate reader and scanned at 765 nm. The calibration curve was made using different concentrations (50 to 200 mg/L) of gallic acid. All measurements were made in triplicate, and Milli- Q water was used as blank.

#### 2.7.2. ABTS

For the ABTS method, the reported method of Re et al. [34] was used. ABTS is a single electron transfer (ET) reaction-based assay. The preparation of PBS was carried out to add 0.8 g of NaCl, 0.02 g of KH_2_PO_4_, 0.115 g of Na_2_HPO_4_, 0.02 g of KCl, and 0.02 g of NaN_3_. Then the solution was filled to 100 mL of Milli Q water. For the ABTS reagent, it was added 38.4 mg of ABTs 1 mM, 6.62 mg of potassium persulfate 2.45 mM, and 10 mL of the solution of PBS. After mixing both solutions, they stirred for 16 h, keep in the dark. To measure the absorbance, the spectrophotometer (Model DR 500, Hach Lange GmbH, Düsseldorf, Germany) was used at 734 nm, a dilution of the initial reagent was needed until it has an absorbance of 0.7 (40 mL of PBS with 3 mL of ABTS solution). The procedure was 20 µL diluted sample, and 2 mL of ABTS solution (absorbance 0.7) were incubated at 30 °C in a water bath for 6 min, then absorbances were read. Trolox was used as standard with concentrations of 5 ppm to 200 ppm. All measurements were made in triplicate. Milli- Q water was used as blank.

#### 2.7.3. Ferric Reducing Antioxidant Power (FRAP)

Based on the procedure of Benzie and Strain [35], the antioxidant capacity of the samples was obtained by the spectrometric method. To prepare the FRAP reagent, three solutions were made. The first one was the acetate buffer 300 mM pH 3.6, for this solution sodium acetate trihydrate, glacial acetic acid, and distilled water were mixed. For the second solution, iron tripyridyltriazine (TPTZ), concentrate HCl and distilled water were used. Then the last solution was FeCl_3_·6H_2_O and Milli-Q water. The three solutions mixed in relation to 10:1:1. FRAP reagent was prepared with 100 mL acetate buffer, 10 mL TPTZ, and 10 mL FeCl_3_·6H_2_O. These compounds were mixed at 30 °C for 30 min. After the incubation, 100 µL of the sample was added to 3 mL FRAP reagent. Trolox was used as a standard with concentrations of 10 to 200 ppm. Finally, absorbance was measured at 593 nm against a blank of the reagent. All measurements were made in triplicate.

#### 2.7.4. α-α-. diphenyl-β-picrylhydrazyl (DPPH)

Based on the procedure described by Brand-Williams [36]. Around 0.0148 g of DPPH was weighed and placed in a volumetric flask (25 mL). Then the volumetric flask was filled to the mark with methanol (mother solution). 1 mL of the solution before mention was placed in a volumetric flask (25 mL) and filled to the mark with methanol (diluted solution). The absorbance was measured in a spectrophotometer (Model DR 500, Hach Lange GmbH, Düsseldorf, Germany). The readings were taken in 2.5 mL cuvette, mixing 75 µL of sample and 3 mL of DPPH diluted solution. The reaction takes place 16 min after mixing reactants. Then absorbances were read at 515 nm. The calibration curve was made using Trolox with concentrations of 5 ppm to 200 ppm. All measurements were made in triplicate.

### 2.8. Minerals Content Analysis

Mineral content was evaluated with digestion in nitric acid (HNO_3_), ICP from Thermo Scientific, model iCAP 6000 Series (Cambridge, England) was used. The following wavelengths (nm) were used, i.e., Ca (396.8), K (766.4), Fe (259.9), Mg (279.5), Mn (257.6), Na (589.5), P (177.4), Cu (324.7), Se (196.0), and Zn (213.8). The digestion of 1 g of sample was carried out during 4 h at a range 85 to 90 °C. The samples were passed through a paper filter (Whatman paper grade 41,110 mm, Item1441110, GE Healthcare Life Sciences, Little Chalfont, UK). The heating was performed with a hot plate (Model Type 2200, Thermo Fisher Scientific, Dubuque, IA, USA). Three samples for each fruit extract were carried out following the same conditions.

### 2.9. Cell Lines and In Vitro Cancer Cell Viability

Normal fibroblast cell line (NIH/3T3), as a control cell line, and four different mammalian cancer cell lines mammary (MCF-7), prostate (PC3), colon (Caco-2) and hepatic (HepG2) were propagated in DMEM-F12 medium containing 10% FBS (Fetal Bovine Serum) (Gibco, Grand Island, NY, USA) and maintained in 5% of CO_2_ atmosphere at 37 °C and 80 % of humidity. The cytotoxicity assay was performed in plates of 96-wells each well was prepared with 100 μL of a cell suspension containing 5 × 10^5^ cells/mL of cancer cells (MCF-7, PC3,Caco-2, and HepG2) and NIH/3T3 after 12 h 100 μL a dilution at 4% of filtrated clarified juice extract in Milli Q water were added to each well in the cell giving a final concentration of 2% of juice in the cell growth media in triplicate (Appendix A). The culture medium without cells was used as a blank. After incubation at 37 °C under 5% CO_2_ for 48 h, 20 μL Cell Titer 96^®^AQueous One Solution Cell Proliferation Assay (Promega, Madison, WI, USA) was used to determine % of cell viability. Absorbance was measured at 490 nm in a microplate reader (Synergy HT, Bio-Tek, Winooski, VT, USA). Cell viability was computed using the average absorbance units obtained from the wells and expressed as a percentage of the untreated cells well.

### 2.10. Phenolic Analysis by HPLC

The determination of phenolic compounds in clarified juices extracts were analyzed by a Perkin Elmer HPLC (Altus 10, Waltham, MA, USA) coupled with a UV-Vis detector. A column Eclipse XDBC18, 5 µm, 150 mm × 4.6 mm (Agilent Technologies, Santa Clara, CA, USA) was used for the analysis. A reversed-phase HPLC was performed, and gradient elution was performed by varying the proportion of solvent A (acidified water with acetic acid pH 2.5) to solvent B (methanol), with a flow rate of 0.8 mL/min. The mobile phase composition started at 100 % solvent A for 3 min, followed by an increase of solvent B to 30% 3 to 8 min, 50% B 8 to 15 min, 30% B 15 to 20 min, and then bring mobile phase composition back to the initial conditions. The reference standards were gallic acid, caffeic acid, coumaric acid, and fumaric acid. The calibration curve range was 10 to 80 mg/L. The aforementioned compounds were selected based on the literature of cacti fruits (*Opuntia ficus Indica and Opuntia littoralis*) [37,38].

## 3. Results and Discussion

### 3.1. Physiochemical Characterization

As is shown in Table 1, chico fruit averaged weight was 77.0 g, equatorial and polar diameters were 4.9 and 6.2 cm, respectively (Figure 1b). Regarding its epicarp, this was higher than its mesocarp, and the percentage of edible fruit was 36.6%. With respect to jiotilla, its average weight was 18.7 g, the edible fruit of jiotilla was 51.9%, and the equatorial and polar diameters were 2.8 and 3.4 cm, respectively. Table 2 shows the proximate analysis of chico fruit and jiotilla. Table 3 shows the total soluble solids, pH, and antioxidants activity in the fruits under study. The pH values were 4.5 (chico fruit) and 4.17 (jiotilla). Regarding the total soluble solids, they were 12.6 (chico fruit) and 7.4 (jiotilla) °Brix.

Comparing the edible percentage, the pitaya (*Stenocereus pruinosus*) fruit has a higher edible percentage (72.5%) [39] than chico fruit and jiotilla. Although the chico fruit (36.6%) and jiotilla (51.9%) have a pulp percentage higher than xoconostle (*Opuntia matudae*) (12% to 18%) [40]. The carbohydrates percentages are higher comparing them with the red pitaya (*Stenocereus pruinosus*) (10.2 ± 0.24%) and orange pitaya (*Stenocereus pruinosus*) (8.5 ± 0.16%) [39], but carbohydrates percentages are lower than presented in other fruits such as Banana (*Musa acuminata*) (22.84%) and Mango (*Mangifera indica*) (17%) [41]. The crude fiber in chico fruit and jiotilla was higher than the percentage in red pitaya (*Stenocereus pruinosus*) (0.67 ± 0.09 %), orange pitaya (0.53 ± 0.02 %) (*Stenocereus pruinosus*) [39]. Furthermore, the protein contents are lower than jackalberry tree fruit (*Diospyros mespiliformis*) (9.28 ± 1.14%) [42], but chico and jiotilla protein percentage are higher than apple (*Malus domestica*) (0.26%) [41].

### 3.2. Antioxidant Capacity of Pulps

Jiotilla (2.32 ± 0.23 mg/g dw) has a higher betacyanin concentration than chico fruit (1.55 ± 0.05 mg/g DW). The concentrations of betaxanthins in chico fruit and jiotilla were 1.46 ± 0.05 and 4.17 ± 0.35 mg/g DW, respectively. The concentration of vitamin C in the chico fruit was 27.19 ± 1.95 mg/100 g FS, while in jiotilla it was not detected. The total antioxidant capacities (Folin-Ciocalteu method) of chico fruit and jiotilla were 113.16 ± 5.82 and 83.40 ± 7.32 mg/100 g FS, respectively. The antioxidant activities determined as ferric reducing-antioxidant power (FRAP) for chico fruit and jiotilla were 0.27 ± 0.002 and 0.315 ± 0.003 mmol/100 g FS, respectively. In chico fruit and jiotilla, the values obtained in ABTS assay were 0.864 ± 0.023 and 0.708 ± 0.124 mmol/100 g FS, respectively. The antioxidant capacity of the fruits understudy was measured by the DPPH assay, which has been employed to measure the antioxidant capacity as well [43]. The free radical scavenging capacities of chico fruit and jiotilla were 34.52 ± 3.43 and 57.17 ± 5.28 mg/100 g FS, respectively. When compared with other cacti fruits, the betacyanins concentrations were lower than the values reported for red pitaya 2.86 ± 0.38 (*Stenocereus pruinosus*) mg/g DW [39] and prickly pear Rojo cenizo (*Opuntia ficus indica*) 5.95 ± 0.21 mg/g DW [44]. Betaxanthins concentration in jiotilla was higher than red pitaya (*Stenocereus Stellatus*) 1.51 ± 0.06 mg/g DW [45] and orange pitaya (*Stenocereus pruinosus*) 2.67 ± 0.27 mg/g DW [39]. Betalains concentration in chico fruit was lower compared with beetroot (*Beta vulgaris*) cultivar little ball in the flesh 3.6 ± 0.2 mg/g DW of betanin and 1.9 ± 0.1 mg/g DW in betaxanthins [46]. Vitamin C concentration in chico fruit, compared with other fruits is lower than guava (*Psidium guajava*) 131 ± 18.2 mg/100 g FS [47]. The fruits for (*Actinidia spp.*) cultivars and citrus species are an excellent source of vitamin C, comparing the (*Actinidia sp.)* species is reported that kiwi (variety Sanuki Gold) has 156 ± 31.2 mg/100 g FS [48] higher than chico fruit concentration. For citrus fruits like lemon (*Citrus limon*), the amount of vitamin C reported is 34 mg/100 g FS [49] and for orange fruit (*Citrus aurantium)* 36.1 mg/100 g FS [47]. It is important to highlight that chico fruits have a similar concentration of vitamin C like lemon juice and orange fruits. When compared total antioxidant capacity (Folin-Ciocalteu method) with some of the fruits recognized to have the highest values, such as raspberry and blackberry, the concentrations were lower. For instance, raspberry (*Rubus idaeus L.*) is reported to present a value of 1489 ± 4.5 mg GAE/100 g FS [50] and for cultivar Aksu Kırmızısı 1040.9 ± 15.9 milligrams of gallic acid per 100 g of FS [51]. Furthermore, a direct comparison of our results with other fruits such as apple red delicious (*Malus domestica*) (73.96 ± 3.52 mg GAE/100 g fw) [52], papaya (*Carcinia papaya Linn*) (54 ± 2.6 mg GAE/100 g FS) [53] and peach (*Prunus persica*) 27.58 ± 1.57 GAE/100 g FS [52] and it is reported for mango (*Mangifera indica L.*) in dry weight 1.64 ± 0.49 mg GAE/g DW [54] (in consideration of this the concentrations of chico fruit and jiotilla were changed to dry weight 9.19 ± 0.47 and 7.09 ± 0.54 mg GAE/g DW, respectively) Jiotilla and chico, represents higher antioxidant activity than fruits mentioned before.

The antioxidant activity determined as FRAP was lower than reported by red rose grape (*Vitis vinifera*) (0.49 ± 0.04 mmol/100 g FS) [55]. Nevertheless, antioxidant activity in chico fruit and jiotilla was higher than the amount in persimmon (*Spyros kaki)* (0.14 ± 0.03 mmol/100 g FS) and duck pear (*Pyrus bretschneideri*) 0.22 ± 0.03 mmol/100 g FS [55]. Dates (*Phoenix dactylifera*) reported amount between 406.61 ± 14.31 and 818.86 ± 21.91 µmol/100 g FS [56], compared with chico fruit and jiotilla. The antioxidant activities are higher in chico fruit (2201.56 ± 22.01 µmol/100 g DW) and jiotilla (2680.91 ± 26.80 µmol/100 g dry weight) than dates (*Phoenix dactylifera*). Comparing the antioxidant activity concentrations (ABTS method) with purple cactus pear (*Opuntia ficus-indica*) (0.61 ± 0.02 mmol/100 g FS) and orange pulp (0.37 ± 0.02 mmol/100 g FS) [6], the antioxidant activities in chico fruit and jiotilla resulted higher than the presented by *Opuntia ficus-indica* pulps. Antioxidant activity compared with fruits from apricot (*Prunus armeniaca*) for the variety Cöloglu, 0.45 ± 0.09 mmol/g FS and the variety Zerdali, 0.37 ± 0.03 mmol/g FS [57], the chico fruit and jiotilla have higher activity than apricot (*Prunus armeniaca*). Concentration units of chico fruit and jiotilla (mmol/100 g FS) were changed to µmol/g fresh sample to compared with other fruits. Chico fruit (8.642 ± 0.204 µmol/g FS) and jiotilla (7.083 ± 1.247 µmol/g FS) antioxidant activity is higher than banana (*Musa acuminata*) 3.44 ± 0.29 µmol/g FS, apple red delicious 4.62 ± 0.03 µmol/g FS and pear (*Pyrussp.*) 4.30 ± 0.06 µmol/g FS [52]. Nevertheless, guava (*Psidium guajava*) 15.18 ± 0.81 µmol/g fs and sweetsop 23.60 ± 0.06 µmol/g fs [52] have a higher concentration than chico fruit and jiotilla. Antioxidant activity (DPPH method) reported in raspberry for Reveille *(Rubus idaeus L.)* (695.58 ± 11.56 mg/100 g FS) [58] is higher than chico fruit and jiotilla. Nevertheless, chico fruit (2.81 ± 0.26 mg/g DW) and jiotilla (4.86 ± 0.45 mg/g DW) antioxidant activities are lower than powder of wild plum tree (*Prunus domestica subsp. Insititia L*.) (26.47 ± 0.19 mg/g DW) [59]. Regarding raspberry extracts of (*Rubus Idaeus L.*), the antioxidant activity has been reported to be 29.0 ± 1.1 µmol/g FW [60] which represents a higher antioxidant activity than both fruits in the study, chico fruit (1.38 ± 0.13 µmol/g FW) and jiotilla (2.28 ± 0.21 µmol/g FS).

### 3.3. Mineral Content Analysis

Table 4 shows mineral content. Magnesium is essential for human metabolism; the enzymes use magnesium as a cofactor. The chico fruit and jiotilla have a magnesium content of 102.26 ± 4.24 mg/100 g and 33.12 ± 2.74, respectively. Regarding the potassium content, the chico fruit has 517.75 ± 16.78 mg/100 g. Comparing magnesium content with the almonds (*Prunus dulcis*) (270 mg/100 g), which represents a high magnesium content [61], chico fruit and jiotilla have lower contents in magnesium than almonds. Likewise, comparing with a cactus, *Opuntia spp*., it is reported for the pulp fruit content of 76 mg/100 g [62], the recommended intake per day is around 400–420 mg/per day [60]. Regarding the potassium content, the chico fruit has 517.75 ± 16.78 mg/100 g, which is a 159 mg/100 g superior to the value presented by the banana (*Musa acuminate*), a fruit with high potassium content, [61]. A comparison with (*Opuntia ficus indica)* fruits authors reported 559 mg of potassium/100 g [62]. It is important to highlight that the recommended intake per day is around 3400 mg potassium/per day [63]. Calcium is important to maintain strong bones, for the calcium content, the chico fruit and jiotilla have 69.99 ± 4.21 and 40.06 ± 1.43 mg/100 g respectively, these values are higher than reported data for banana (*Musa acuminate*) (26 mg/100 g), the pineapple (*Ananas comosus (L.) Merr*) (21 mg/100 g), papaya (*Carcinia papaya Linn*) (16 mg/100 g) and pitahaya (*Hylocereus undatus*) (31 mg/100 g) [64]. Zinc mineral also is important for children’s growth, the chico fruit has 2.46 ± 0.65 mg/100 g, comparing this with reported values of different fruits, that the mango (*Mangifera indica*) has 0.14 mg/100 g, the papaya (*Carica papaya L.)* has 0.09 mg/100 g [64]. Zinc content is higher than fruits before mentioned. The zinc concentration in chico fruit is similar to borojo (*Borojoa sorbilis*) (2.47 mg/100 g) [64]. Nevertheless, the content of chico in zinc is lower than camajon fruit (*Sterculia apetala*) fruit 5.70 mg/100 g (our knowledge the fruit with the highest concentration of zinc) [64].

### 3.4. Antioxidant Capacity of Clarified Juice Extracts

Clarification is a procedure that removes the solids from a liquid, usually a beverage like wine. The concentration of betacyanins in clarified juice of chico fruit and jiotilla was 1.38 ± 0.01 and 2.93 ± 0.04 mg/g DW. Regarding the betaxanthin concentrations, in chico fruit and jiotilla clarified juice extracts were 1.16 ± 0.01 and 2.52 ± 0.35 mg/g DW. The total antioxidant capacity (Folin–Ciocalteu method) in chico fruit and jiotilla clarified juices were 129.47 ± 1.12 and 195.39 ± 7.82 mg/100 g FS. The antioxidant capacities of clarified juices are shown in Table 5 by methods FRAP, ABTS, and DPPH.

In clarification procedure some antioxidant capacity could be lost [65]. This phenomenon has been reported for green tea (*Camellia sinensis*) [66] and apple juice, where the clarification process reduces 2.5 times its antioxidant capacity [67]. However, in this work, the clarification increased antioxidant capacity. Similar results were observed in freeze-dried cherry laurels (*Prunus laurocerasus*) [68]. The increase in antioxidant capacity after centrifugation in liquids derived from vegetal material is probably due to the fact that antioxidants (polyphenols and betalains) are water-soluble and they are released from the pulp. The concentration of betacyanins in clarified juice of jiotilla (345.52 ± 5.04 µg/g FS) was higher than prickly pear juice (*Opuntia robusta*) betacyanins 300.5 ± 8.8 µg/g FS [7]. Contrary to the concentration found in clarified juice of chico fruit (151.6 ± 1.3 µg/g FS). Furthermore, this clarified juice is lower than the concentration reported for juice of *Opuntia rastrera* 152.6 ± 5.4 µg/g FS [7]. Regarding the betaxanthin concentration, in jiotilla clarified juice (296.8 ± 4.2 µg/g FS) is higher than prickly pear juice (*Opuntia robusta*) 189.9 ± 7.3 µg/g FS [7]. Also, chico fruit clarified juice has a higher betaxanthin concentration (127.5 ± 1.2 µg/g FS) than juice of *Opuntia rastrera* (86.2 ± 22.3 µg/g FS) [7]. Finally, the total antioxidant capacity (Folin-Ciocalteu method) in chico fruit (1294.76 ± 0.01 mg/100 g FS) and jiotilla (1953.39 ± 0.0701 mg/100 g FS) clarified juices are higher than prickly pear juice of Duraznillo rojo (*Opuntia leucotricha*) 226.3 ± 26.4 µg GA/g FS [7]. Comparing with other juices from commonly edible fruits, antioxidant activity in jiotilla juice clarified (3406.6 ± 539.6 µmol/100 mL) is higher than pomegranate (*Punica granatum)* 3281.8 µmol/100 mL and aronia (*Aronia melanocarpa*) and 3277.9 µmol/100 mL juice with FRAP method [69].While in chico fruit juice clarified (665.9 ± 18.1 µmol/100 mL) antioxidant activity is higher than orange juice 203.4 µmol/100 mL [69]. Nevertheless, chico fruit (2983.9 ± 73.4 µmol/100 mL) and jiotilla (2462.6 ± 174.7 µmol/100 mL) juices have lower antioxidant activity than pomegranate 4537.3 µmol/100 mL and aronia 4261.8 µmol/100 mL juices in the ABTS method [69]. Chico fruit 319.7 ± 20.4 µmol/100 mL and Jiotilla 1122.2 ± 30.4 µmol/100 mL have lower antioxidant activity than pomegranate (3138.3 µmol/100 mL) juice in DPPH [69]. Clarified juices extract of chico has a higher antioxidant activity than clarified juice extract of jiotilla, as evident by the ABTS method. This method reacts with any hydroxylated aromatics independently of their real antioxidant activity, including OH-groups, which do not contribute to antioxidant activity [69].

### 3.5. Cytotoxicity of Clarified Juice Extracts

**Figure 3:**
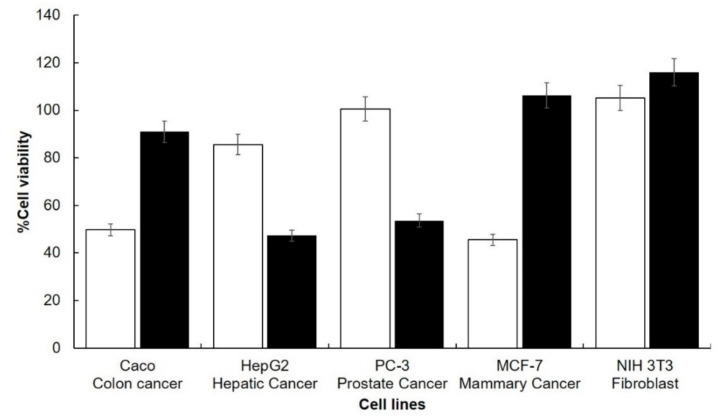
Effect of clarified juices extracts of chico fruit (white) and jiotilla (black) at 2% on the cell viability. The error bars are standard deviations.

The cytotoxicity of the juice extracts at 2% is shown in Figure 3. NIH/3T3 cell line was used as a control in order to screen the cytotoxicity of the clarified juices extract of jiotilla and chico on normal cells, as demonstrated in Figure 3, they were not cytotoxic in normal cell lines compared to cancer cell lines. The synergetic effect between betalains and phenolic compounds of cacti juice have been reported with benefits such as anticancer [7,8] and anticlastogenic effects [2]. Chico fruit and jiotilla showed a high concentration of betalains and phenolic compounds respect to different fruits. This contain could be responsible for the antioxidant capacity and cytotoxicity. Furthermore, phenolic acids were analyzed in order to identify the antiproliferative compounds. No cytotoxicity was observed in both juices in cell line NIH/3T3, used as a normal cell in order to evaluate the cytotoxicity effect of the juices relative to cancer cells, these values are congruent compared with other cacti fruits such as red pitahaya (*Hylocereus polyrhizus*) in normal human cell lines (HEK-293/human embryonic kidney and TPH-1/hummonocytes in the concentration of 0.39 to 0.78 mg/mL at 48 h) [4], and *Opuntia spp* Cardón (NIH/3T3 in concentration of 0.5% juice at 48 h) [7]. Chico fruit juice (49.7 ± 0.01%) inhibited the CaCo-2 growth and showed similar cell viability as gavia *Opuntia robusta* 52.50 ± 12.60% [7]. Cacti fruits have been tested in other colorectal cancer cell line HT29 and produced cytotoxicity an effective dose values (ED50 5.8 ± 1.0% *v*/*v* at 96 h) [8]. Cytotoxicity of chico fruit juice was observed (45.56 ± 0.05) in MCF-7, the significant effect was compared to *Opuntia rastrera* 75.40 ± 8.26% [7]. Other foods with a high concentration of betalains such as Beta vulgaris extract was cytotoxic in MCF-7 with IC50 value of 70 µg/mL, and cytotoxicity increase with the combination of Beta vulgaris extract and silver nanoparticles (IC50 47.6 µg/mL) [70]. The combination of chico fruit juice and silver nanoparticles could generate the same effect mentioned above. Jiotilla juice showed a higher value of cytotoxicity (47.31 ± 0.03%) in HepG2 than *Opuntia rastrera* 78.90 ± 9.00% [7]. Betanin from beetroot presented a 49% inhibition of HepG2 cell proliferation [71]. Other benefits that cacti juices have shown are hepatoprotective effect in-vitro and in-vivo [3,72]. Jiotilla juice (53.65 ± 0.04%) diminished the cell viability in PC-3 than moradillo *Opuntia violaceae* 61.20 ± 5.30% [7]. Extract of *Beta vulgaris L*. also has cytotoxicity in PC-3 (IC50 316.0 ± 2.1 µg/mL) [73].

### 3.6. Phenolic Analysis by HPLC

Table 6 shows phenolic acid concentrations of chico fruit and jiotilla that compounds have cytotoxicity in cancer cell lines. *p*-Coumaric acid, a hydroxy derivative of cinnamic acid, is a compound that has a significant antiradical scavenging effect [74]. *p*-Coumaric acid is believed to reduce the risk of stomach cancer by reducing the formation of carcinogenic nitrosamines [75,76]. *p*-Coumaric acid has therapeutic benefits against cancer cell lines (Caco-2) [77]. The *p*-Coumaric acid-induced apoptosis in colon cancer cells (HTC-15) through the ROS-mitochondrial pathway [78]. Furthermore, *p*-Coumaric acid is shown to possess anti-inflammatory, anti-ulcer, anti-cancer, and anti-mutagenic properties [79,80]. Gallic acid, a polyhydroxy phenolic compound that can be found in green tea, grapes, strawberries, and bananas [81]. Gallic acid has demonstrated the potential anticancer activity in vivo and in vitro [82,83]. The anticancer activity of Gallic acid has been reported in various cancer cells, including human ovarian cancer cells (HeLa), leukemia cell lines (C121) [84,85]. It was proven that the anticancer effect of Gallic acid is due to its ability to inhibit cell proliferation and to induce apoptosis [86,87]. Caffeic acid, the primary representative of hydroxycinnamic acids and phenolic acid in general, is widely distributed in plants [88]. Caffeic acid has an action against cervical (HeLa), mammary gland adenocarcinomas (MDA-MB-231), lymphoblastic leukemia (MOLT-3) [88], but it is not cytotoxic to healthy cells [89]. Further, caffeic acid treatment altered the mitochondrial membrane potential on HT-1080 human fibrosarcoma cell line. Other compounds that affect cancer cell lines are the betacyanins, and the main structure is betanin. Kapadia et al. found that betanin has cytotoxic properties in PC-3 cells [73] and Lee et al. observed that the same compound has cytotoxicity in HepG2 [71]. Clarified juice extract of jiotilla shows cytotoxicity in PC-3 and HepG2, this clarified juice has 2.12 times more betacyanins than clarified juice extracts of chico. Vitamin C promotes apoptosis in MCF-7 [90], although the concentration of vitamin C was not analyzed in the clarified juices, it was detected in the chico fruit (27.19 ± 1.95 mg/100 g FS). Probably, it is the main compound in clarified juice extract of chico that produces the cytotoxicity in MCF-7. In this work was identified the main compounds that have been reported in cacti fruits (betalains, p-coumaric acid, caffeic acid, ferulic acid, gallic acid) [37,38,91]. There is a synergy between betalains and phenolic compounds in antioxidant activity and cytotoxicity.

## 4. Conclusions

The presence of a large variety of compounds of high-value in several fruits has been recognized in several studies. In this research, we investigated and demonstrated the nutritional characteristics of two desert fruits, i.e., chico fruit and jiotillain pulp and their clarified juice extracts. Chico fruit was seen to be an excellent resource of vitamin C, potassium, and zinc. Meanwhile, in jiotilla, betacyanins and betaxanthins were the main compounds that deserve to be highlighted, as their concentration was high as compared to earlier reported sources. All these results measured in the pulp of both fruits. On one hand, the determination of the cytotoxicity produced by clarified juice of both fruits in normal cell lines was absent. On the other hand, clarified juice extract of chico fruit showed cytotoxicity in CaCo and MCF-7. Regarding the clarified juice extract of jiotilla, the cytotoxicity activity was showing HepG2 and PC-3 cell lines. For both fruits, in the clarified juice and pulp, the nutritional profiles resulted are high and, in some instances, similar compared with some other and more common edible fruits. Overall, this work increased the knowledge of other different sources of nutrients that can be used to feed the population.

## Figures and Tables

**Table 1:** Physical characterization of the collected plant samples.

Parameter	Chico Fruit	Jiotilla
Equatorial diameter (cm)	4.87 ± 0.28 ^1^	2.81 ± 0.29
Polar diameter (cm)	6.25 ± 0.11	3.48 ± 0.36
Fruit weight (g)	77.0 ± 14.5	18.7 ± 3.12
Mesocarp weight (g)	28.0 ± 9.4	9.21 ± 1.95
Epicarp weight (g)	48.4 ± 10.8	8.53 ± 1.53

^1^ Results are shown as average ± standard deviation.

**Table 2:** General proximate analysis of the collected plant samples.

Parameter	Chico Fruit	Jiotilla
Moisture (%)	71.32 ± 0.46 ^1^	85.21 ± 0.54
Carbohydrates (%)	18.84 ± 0.29	12.75 ± 0.19
Protein (%)	4.75 ± 0.10	1.025 ± 0.02
Ethereal extract (%)	1.87 ± 0.06	0.48 ± 0.01
Crude fiber (%)	2.52 ± 0.09	1.31 ± 0.04
Ash (%)	0.7 ± 0.03	0.53 ± 0.02

^1^ Results are shown as average ± standard deviation.

**Table 3:** Chemical characterization of the collected plant samples.

Parameter	Chico Fruit	Jiotilla
Total soluble solids °Brix	12.6	7.4
pH	4.51	4.17
Betacyanins (mg/g DW)	1.55 ± 0.05 ^1^	2.32 ± 0.23
Betaxanthins (mg/g DW)	1.46 ± 0.05	4.17 ± 0.35
Vitamin C (mg/100g FS) ^2^	27.19 ± 1.95	Not detected
Folin-Ciocalteu method (mg/100g FS) ^3^	113.16 ± 5.82	83.40 ± 7.32
ABTS (mmol/100 g FS) ^4^	0.864 ± 0.020	0.708 ± 0.124
FRAP (mmol/100g FS) ^4^	0.27 ± 0.002	0.315 ± 0.003
DPPH (µmol/g FS) ^4^	34.52 ± 3.43	57.17 ± 5.28

^1^ Results are shown as average ± standard deviation. ^2^ Ascorbic acid. ^3^ Gallic acid. ^4^ Trolox were used in calibration curves. DW: dry weight; FS: fresh sample.

**Table 4:** Mineral content analysis profile of chico and jiotilla samples.

Parameter	Chico Fruit	Jiotilla
Ca (mg/100 g)	69.99 ± 4.21 ^1^	40.06 ± 1.43
K (mg/100 g)	517.75 ± 16.78	295.43 ± 48.91
Fe (mg/100 g)	2.30 ± 0.80	0.635 ± 0.26
Mg (mg/100 g)	102.26 ± 4.84	33.12 ± 2.74
Mn (mg/100 g)	1.07 ± 0.03	0.69 ± 0.06
Na (mg/100 g)	91.28 ± 22.57	1.90 ± 0.27
P (mg/100 g)	57.78 ± 2.01	16.72 ± 1.49
Cu (mg/100 g)	<2.71 ± 0.02	<0.35
Se (mg/100 g)	<1.41 ± 0.01	<0.1825
Zn (mg/100 g)	2.46 ± 0.65	0.38 ± 0.05

^1^ Results are shown as average ± standard deviation.

**Table 5:** Antioxidant activity clarified juices extracts.

Parameter	Chico	Jiotilla
Betacyanins (mg/g DW)	1.38 ± 0.01 ^1^	2.93 ± 0.04
Betaxanthins (mg/g DW)	1.16 ± 0.01	2.52 ± 0.35
Folin-Ciocalteu method (mg/100g FS) ^2^	129.47 ± 1.12	195.39 ± 7.82
FRAP (mmol/100g FS) ^3^	0.665 ± 0.018	3.40 ± 0.539
ABTS (mmol/100 g FS) ^3^	2.98 ± 0.07	2.46 ± 0.17
DPPH (mg/100 g FS) ^3^	80.01 ± 5.10	280.88 ± 7.62

^1^ Results are shown as average ± standard deviation. ^2^ Gallic acid. ^3^ Trolox were used in calibration curves. DW: dry weight; FS: fresh sample.

**Table 6:** Amount of the polyphenolic (mg/100 g fresh sample).

Parameter	Chico Fruit	Jiotilla
*p*-Coumaric acid	0.32 ± 0.04	N.D.
Gallic acid	0.50 ± 0.01	1.02 ± 0.01
Caffeic acid	0.3 ± 0.00	0.08 ± 0.00
Fumaric acid	N.D.	N.D.

N.D.—Not detected.

**Figure A1:**
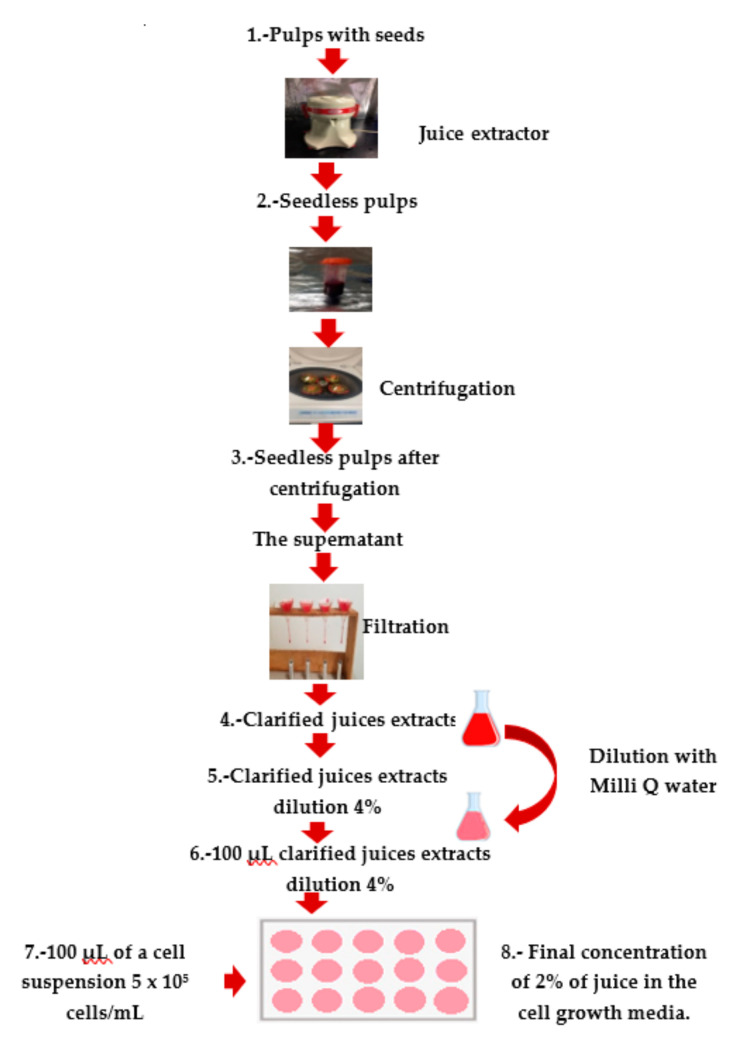
Dilution of clarified juice extract.
